# Identification of gut dysbiosis in axial spondyloarthritis patients and improvement of experimental ankylosing spondyloarthritis by microbiome-derived butyrate with immune-modulating function

**DOI:** 10.3389/fimmu.2023.1096565

**Published:** 2023-04-18

**Authors:** Hong Ki Min, Hyun Sik Na, JooYeon Jhun, Seon-Yeong Lee, Sun Shim Choi, Go Eun Park, Jeong Su Lee, In Gyu Um, Seung Yoon Lee, Hochan Seo, Tae-Seop Shin, Yoon-Keun Kim, Jennifer Jooha Lee, Seung-Ki Kwok, Mi-La Cho, Sung-Hwan Park

**Affiliations:** ^1^ Division of Rheumatology, Department of Internal Medicine, Konkuk University Medical Center, Seoul, Republic of Korea; ^2^ The Rheumatism Research Center, Catholic Research Institute of Medical Science, College of Medicine, The Catholic University of Korea, Seoul, Republic of Korea; ^3^ Lab of Translational ImmunoMedicine, Catholic Research Institute of Medical Science, College of Medicine, The Catholic University of Korea, Seoul, Republic of Korea; ^4^ Department of Medical Life Sciences, College of Medicine, The Catholic University of Korea, Seoul, Republic of Korea; ^5^ Department of Biomedicine and Health Sciences, College of Medicine, The Catholic University of Korea, Seoul, Republic of Korea; ^6^ Division of Biomedical Convergence, College of Biomedical Science, Institute of Bioscience and Biotechnology, Kangwon National University, Chuncheon, Republic of Korea; ^7^ MD Healthcare Inc., Seoul, Republic of Korea; ^8^ Division of Rheumatology, Department of Internal Medicine, Seoul St. Mary’s Hospital, College of Medicine, The Catholic University of Korea, Seoul, Republic of Korea

**Keywords:** spondyloarthropathies, dysbiosis, gastrointestinal microbiome, *Faecalibacterium prausnitzii*, butyrate

## Abstract

**Introduction:**

Dysbiosis is an environmental factor that affects the induction of axial spondyloarthritis (axSpA) pathogenesis. In the present study, we investigated differences in the gut microbiota of patients with axSpA and revealed an association between specific gut microbiota and their metabolites, and SpA pathogenesis.

**Method:**

Using 16S rRNA sequencing data derived from feces samples of 33 axSpA patients and 20 healthy controls (HCs), we examined the compositions of their gut microbiomes.

**Results:**

As a result, axSpA patients were found to have decreased α-diversity compared to HCs, indicating that axSpA patients have less diverse microbiomes. In particular, at the species level, *Bacteroides* and *Streptococcus* were more abundant in axSpA patients than in HCs, whereas *Faecalibacterium (F). prausnitzii*, a butyrate-producing bacteria, was more abundant in HCs. Thus, we decided to investigate whether *F. prausnitzii* was associated with health conditions by inoculating *F. prausnitzii* (0.1, 1, and 10 μg/mL) or by administrating butyrate (0.5 mM) into CD4^+^ T cells derived from axSpA patients. The levels of IL-17A and IL-10 in the CD4^+^ T cell culture media were then measured. We also assessed osteoclast formation by administrating butyrate to the axSpA-derived peripheral blood mononuclear cells. The CD4^+^ IL-17A^+^ T cell differentiation, IL-17A levels were decreased, whereas IL-10 was increased by *F. prausnitzii* inoculation. Butyrate reduced CD4^+^ IL-17A^+^ T cell differentiation and osteoclastogenesis.

**Discussion:**

We found that CD4^+^ IL-17A^+^ T cell polarization was reduced, when *F. prausnitzii* or butyrate were introduced into curdlan-induced SpA mice or CD4^+^ T cells of axSpA patient. Consistently, butyrate treatment was associated with the reduction of arthritis scores and inflammation levels in SpA mice. Taken together, we concluded that the reduced abundance of butyrate-producing microbes, particularly *F. prausnitzii*, may be associated with axSpA pathogenesis.

## Introduction

Axial spondyloarthritis (axSpA) is a type of systemic inflammatory arthritis, which presents with joint and extra-articular symptoms such as inflammatory bowel disease (1). The most well-known marker gene for axSpA is HLA-B27, and several other genes are known to be associated with axSpA pathogenesis (2). In addition, environmental factors are known to play a role in inducing axSpA (1). For example, exposure to specific pathogens *via* the gastrointestinal or genitourinary tracts increases the risk of axSpA (1). With regard to the immune system, evidence suggests that the interleukin (IL)-23 and IL-17 axis is a cornerstone pathological component of axSpA (3). Although various cells such as mucosa-associated invariant T cells, gamma-delta T cells, and type 3 innate lymphoid cells are responsible for producing IL-17, type 17 helper T cells (Th17) are the key pathogenic cell type involved in axSpA (3). Circulating Th17 are increased in patients with ankylosing spondylitis (AS), a prototype of axSpA (4), but this can be effectively attenuated with tumor necrosis factor (TNF) inhibitors (5).

Since fecal microbiota transplantation were shown to exhibit therapeutic effects on *Clostridioides difficile* infection, the role of gut dysbiosis on the pathogenesis of inflammatory or autoimmune diseases has become the focus of much research (6, 7). The first study on gut dysbiosis in patients with SpA was reported by Stoll et al. (8) Thereafter, several studies revealed the altered composition of the gut microbiome in axSpA patients, although these studies differed according to several factors such as sample site, age, sex, and ethnicity (9). The method of sampling gut microbiota is varied (10), but patient self-collection of feces is still the most widely used method. In addition, gut dysbiosis in Korean axSpA patients has not yet been evaluated.

Short-chain fatty acids (SCFAs) such as butyrate, acetate, and propionate are metabolites of gut microbiota (11). Recent studies have demonstrated an association between the gut microbiome, its metabolites, and inflammatory diseases (12). *Faecalibacterium prausnitzii* (*F. prausnitzii*) produces butyrate, which is known to regulate CD4^+^ T cell differentiation in animal models of colitis (13). In an HLA-B27/β2 microglobulin transgenic rat model of spondyloarthritis, alterations in gut metabolites and administration of propionates attenuated inflammatory cytokine expression (14).

In this study, we compared the gut microbiome of axSpA patients and healthy controls (HCs) in Korea. Furthermore, the immune modulatory role of specific microbiota and their metabolites on helper T cell differentiation and osteoclatogenesis in axSpA patient-derived cells was evaluated. Finally, the *in vivo* effects of butyrate treatment were evaluated in a SpA mouse model.

## Materials and methods

### Patients

Feces from axSpA patients and HCs was collected from two university based tertiary hospitals, Seoul Saint Mary’s Hospital and Konkuk University Medical Center (Seoul, South Korea). The inclusion criteria for axSpA patients were as follows: 1) Fulfillment of the 2009 Assessment of SpondyloArthritis International Society classification criteria for axSpA (15), and 2) aged > 19 years old. AxSpA patients with another autoimmune diseases, inflammatory arthritis, active infections, malignancy, or pregnancy were excluded. Baseline demographics, laboratory results, medications, and disease history were collected. Disease-associated parameters such as Ankylosing Spondylitis Disease Activity Index Score with C-reactive protein (ASDAS-CRP) (16), Bath Ankylosing Spondylitis Disease Activity Index (BASDAI) (17), Bath Ankylosing Spondylitis Functional Index (18), and a patient global assessment were recorded when the fecal samples were collected. Peripheral blood samples were also obtained. The present study was conducted in accordance with the Declaration of Helsinki and Good Clinical Practice guidelines. The experimental protocol was approved by the Institutional Review Boards of Seoul Saint Mary’s Hospital (KC18TESI0627) and Konkuk University Medical Center (KUH 2019–08–015). Written informed consent was obtained from each participant prior to enrollment.

### Isolation and stimulation of CD4+ T cells

Human peripheral blood mononuclear cells (PBMCs) were collected from heparinized blood by standard Ficoll-Paque density gradient centrifugation (GE Healthcare Biosciences, Uppsala, Sweden). Human PBMCs were incubated with CD4-coated magnetic beads (Miltenyi Biotec, Bergisch Gladbach, Germany) and isolated on MACS separation columns (Miltenyi Biotec). CD4^+^ T cells (5 x 10^5^ cells) were seeded into 48-well plates (Nunc, Rosklide, Denmark). Th17 cell-polarizing conditions, the sorted CD4^+^ T cells were stimulated with plate-bound anti-CD3 (0.5 μg/mL), anti-CD28 (1 μg/mL), anti-IFN-γ (2 μg/mL), anti-IL-4 (2 μg/mL), TGF-β (2 ng/mL), and IL-6 (20 ng/mL) with F. prausnitzii (1 or 10 μg/mL) or butyrate (0.5 or 1 mM) for 72 h to investigate the effect of F. prausnitzii or butyrate on CD4+ T cell differentiation.

### Flow cytometry analysis

Cytokine expression was analyzed *via* intracellular staining with the following antibodies: Alexa-647-conjugated anti-IL-17 (catalog number 560437) and Alexa-700-conjugated anti-CD4 (catalog number 557922) (BD Biosciences, San Diego, CA, USA). Before staining, cells were stimulated for 4 h with phorbol myristate and ionomycin in the presence of GolgiStop (BD Biosciences, San Diego, CA, USA). To analyze regulatory T cell (Treg) populations, PBMCs were stained with Alexa-700-conjugated anti-CD4, PerCP-Cy5.5-conjugated CD25 (catalog number 560503), and Alexa-647-conjugated FoxP3 (catalog number 561184) (BD Biosciences, San Diego, CA, USA). Th17 and Treg populations were determined using specific antibodies. After surface staining for 30 min, the cells were permeabilized with Cytofix/Cytoperm solution (BD Biosciences, San Diego, CA, USA). Thereafter, the cells were intracellularly stained with fluorescent antibodies. Flow cytometry was performed with the aid of a cytoFLEX Flow Cytometer (Beckman Coulter, Brea, CA, USA), and flow cytometry data were analyzed using FlowJo (Tree Star, Ashland, OR, USA).

### Enzyme-linked immunosorbent assay (ELISA) of IL-10 and IL-17A in culture media

Briefly, a 96-well plate (Eppendorf, Hamburg, Germany) was coated with monoclonal antibodies against IL-17A (DY317; R&D systems, Minneapolis, MN, USA), and IL-10 (DY217B; R&D systems) at 4°C overnight. After blocking with a phosphate-buffered saline (PBS)/1% bovine serum albumin/0.05% Tween 20 solution for 2 h at room temperature (22–25°C), the test samples and standards (recombinant IL-10 and IL-17A) were added to the 96-well plate and incubated at room temperature for another 2 h. The plates were washed four times with PBS and Tween 20 and then incubated with biotinylated human monoclonal antibodies against IL-10 and IL-17A for 2 h at room temperature. After washing, streptavidin-alkaline phosphatase-horseradish peroxidase conjugate (Sigma, St Louis, MA, USA) was added to the wells for 30 min, followed by incubation with 1 mg/mL p-nitrophenyl phosphate (Sigma, St Louis, MA, USA) dissolved in diethanolamine (Sigma, St Louis, MA, USA) to develop the color reaction. The reaction was stopped by the addition of 1 M NaOH, and the optical density of each well was measured at 405 nm.

### Human *in vitro* osteoclastogenesis

PBMCs obtained from axSpA patients (N = 4) were separated from buffy coats using Ficoll-Hypaque. Red blood cell-free cells were seeded onto 24-well plates at 5 x 10^5^ cells/well and incubated at 37°C for 2 h to separate the floating and adherent cells. The adherent cells were washed with sterile PBS. The preosteoclasts were further cultured in the presences of 10 ng/mL macrophage colony-stimulating factor (M-CSF), 100 ng/mL receptor activator of RANKL (PepproTech, London, UK), and butyrate (0.5 mM) for 4 days to generate osteoclasts. The medium was changed every 2 days and osteoclasts were generated after 8–10 days.

### Animal model

Twelve-week-old male SKG mice weighing 20–25 g at the start of the experiment were purchased from CLEA Japan Inc. (Tokyo, Japan). A maximum of three animals per cage were housed in a room with temperature (20–26°C) and light (12-h light-dark cycle) controlled conditions. The mice had free access to a gamma-ray-sterilized diet (TD 2018S; Harlan Laboratories, Indianapolis, IN, USA) and autoclaved R/O water. All animal research was conducted in accordance with the Laboratory Animals Welfare Act, the Guide for the Care and Use of Laboratory Animals, and the Guidelines and Policies for Rodent Experimentation provided by the Institutional Animal Care and Use Committee (IACUC) at the school of medicine of The Catholic University of Korea (Approval number: CUMS–2020–0312–03). The IACUC and Department of Laboratory Animals at the Catholic University of Korea, Songeui Campus, was accredited by the Korean Excellence Animal Laboratory Facility in accordance with the Korean Food and Drug Administration in 2017 and full accreditation by the Association for Assessment and Accreditation of Laboratory Animal Care International was acquired in 2018.

### Induction of spondyloarthritis and treatment with sodium butyrate

SKG mice were injected with 3 mg of curdlan (Wako Chemicals, Richmond, USA) dissolved in a 200-μl volume using a 26.5-G needle *via* intraperitoneal injection and oral administration. After 1 week, animals were equally divided into groups based on the mean values ​​of their arthritis score. Then, 0.9% saline and sodium butyrate (100 mg/kg) were administered orally once a day.

### *In vivo* arthritis scoring

The arthritis score index for disease severity was defined as follows: 0.1, a single site of interphalangeal swelling; 0.5, moderate swelling of the wrist or ankle; and 1.0, severe swelling of the wrist or ankle. The maximum possible score per mouse was 5.8. The scoring was carried out by two independent observers without knowledge of the experimental and control groups.

### Histopathological analysis

Tissues were collected from each group 15 weeks after the induction of spondyloarthritis. Tissues were fixed in 10% formalin solution, decalcified using Calci-Clear (National Diagnostics, Atlanta, USA), and embedded in paraffin. Sections of 4- to 5-μm thickness were cut, dewaxed using xylene, dehydrated through an alcohol gradient, and then stained with hematoxylin and eosin (H&E) and safranin O.

### Immunohistochemistry

Paraffin-embedded sections were incubated at 4°C with the following primary monoclonal antibodies: anti-IL-1β (catalog number NB600-633) (Novus Biologicals, Littleton, CO, USA), anti-IL-17 (catalog number ab91649), and anti-TNF-α (catalog number ab6671) (Abcam, Cambridge, UK). The samples were then incubated with their respective secondary biotinylated antibodies, followed by a 30-min incubation with a streptavidin-peroxidase complex. The reaction product was developed using 3, 3-diaminobenzidine chromogen (Dako, Santa Clara, CA, USA).

### Histopathology scoring

The H&E-stained joint samples were scored for inflammation, bone erosion, and cartilage damage. Inflammation was scored according to the following criteria: 0, no inflammation; 1, slight thickening of the lining layer or some infiltrating cells in the underlying layer; 2, slight thickening of the lining layer plus some infiltrating cells in the underlying layer; 3, thickening of the lining layer, an influx of cells in the underlying layer, and the presence of cells in the synovial space; and 4, the synovium highly infiltrated with many inflammatory cells (19). Cartilage damage was evaluated by staining with safranin O and the extent of damage was scored as follows: 0, no destruction; 1, minimal erosion (limited to single spots); 2, slight-to-moderate erosion in a limited area; 3, more extensive erosion; and 4, general destruction. In addition, spinal samples were scored for the condition of the nucleus pulposus (NP) and annulus fibrosus (AF). The NP was scored as follows: 0, normal; 1, slightly condensed; 2, moderately condensed (fibrosis); 3, severely condensed (scattering cells); 4, mild replacement by fibrous cartilaginous tissue; and 5, moderately or severely replaced by fibrous cartilaginous tissue. The AF was scored as follows: 0, normal; 1, serpentine; 2, mildly reversed contour (internally convex); 3, thickened with central ingrowth or rupture; 4, vague borderline with NP; and 5, indistinct.

### Flow cytometric analysis (mouse)

Cell pellets were prepared from the spleens of spondyloarthritic SKG mice. To examine the population of T helper cells, the cells were stained with PerCP-conjugated anti-CD4 (catalog number 45-0042-82) and APC-conjugated anti-CD25 (catalog number 102012) antibodies (eBioscience; Biolegend, San Diego, CA, USA), and then permeabilized and fixed with CytoFix/CytoPerm (BD Biosciences, San Diego, CA, USA) according to the manufacturer’s instructions. The cells were then further stained with PE-conjugated anti-FoxP3 (catalog number 12-5773-82), APC-conjugated anti-interferon (IFN) (catalog number 505810), and FITC-conjugated anti–IL-17 (catalog number 11-7177-81) (eBioscience; Biolegend, San diego, CA, USA).

### *In vitro* assay of Th17 stimulation

Spleen samples were collected from normal SKG mice. Splenocytes were incubated with CD4-coated magnetic beads and isolated using magnet-activated cell sorting separation columns (Miltenyi Biotech, Auburn, CA, USA). To establish Th17 cell-polarizing conditions, the sorted CD4^+^ T cells were stimulated with plate-bound anti-CD3 (0.5 μg/mL), anti-CD28 (1 μg/mL), anti-IFN-γ (2 μg/mL), anti-IL-4 (2 μg/mL), TGF-β (2 ng/mL), and IL-6 (20 ng/mL) for 72 h.

### Enzyme-linked immunosorbent assay (mouse)

The amounts of IL-17 and TNF-α in culture supernatants from mouse cells were measured by a sandwich ELISA (R&D Systems, Minneapolis, MN, USA). Horseradish peroxidase-avidin was used for color development. The absorbance was determined at a wavelength of 450 nm on an Multiskan SkyHigh Microplate Spectrophotometer (Thermo Fisher, Waltham, MA, USA).

### Generation of 16S rRNA sequencing data

Using feces samples from 33 axSpA patients and 20 HCs, the V3-V4 amplicon sequencing of 16S rRNA data was performed with an Illumina MiSeq reagent kit v3 (2 x 300 bp, Illumina, USA). The PCR primers, i.e., forward (CCTACGGGNGGCWGCAG) and reverse (GACTACHVGGGTATCTAATCC), were designed from the hypervariable regions (V3-V4) of 16S rRNA. PCR was performed using the following conditions: 95°C for 3min, followed by 25 cycles of denaturation at 95°C for 30 sec, primer annealing at 55°C for 30 sec, and extension at 72°C for 30 sec, with a final elongation at 72°C for 5 min. Sequencing libraries were then constructed using a TruSeq^®^ DNA PCR-Free Sample Preparation Kit (Illumina, USA), TruSeq^®^ Nextera XT index primer (Illumina), and 2x KAPA HiFi HotStart ReadyMix (Roche, Basal, Switzerland) using the purified PCR products. Subsequently, paired-end reads were generated by sequencing on the MiSeq platform after determining the quality of the library with the Tapestation 4200 platform (Agilent Technologies, Santa Clara, CA, USA) and a Qubit Fluorometer (Thermo Fisher, Waltham, MA, USA). Metagenome analysis data was submitted to National Center for Biotechnology Information BioProject (PRJNA863406).

### Analysis of the compositions of microbiomes

Microbiomes were identified by the bioinformatics procedures described in Choi et al. (20) After trimming the low-quality sequences and adapter sequences using Cutadapt (21) and Trimmomatic (22), the paired-end reads were assembled using the PEAR (23) tool. An average of 46,560 high-quality reads per sample (median: 29,385 bp; range: 11073–179248 bp) were obtained, in which the average length and quality score were 413.3 bp and 37.24 bp, respectively. The 16S rRNA sequencing data were processed using QIIME2 (ver. 2021.8) (24). Single-end reads for 53 microbiomes from axSpA and HC samples were imported into QIIME2. Sequence quality control and feature table construction were completed using the Divisive Amplicon Denoising Algorithm 2 (DADA2) QIIME2 plugin. The cleaned sequences were then used for the identification of operational taxonomic units (OTUs) with singleton OTUs excluded. Information on the taxonomic hierarchy from phylum to species of each sample was defined by aligning the sequence to the SILVA reference database (release 138) (25) by the pretrained naive Bayes classifier (26) at a 99% minimum similarity level. Only taxa with at least 0.1% relative abundance in each group were used to generate a taxonomic profile graph based on the final OTU table. Linear discriminant analysis effect size (LEfSe) (27) was applied for investigating the compositional information of the microbiomes differentially enriched between axSpA and HC samples.

### Analysis of α- and β-diversity

Using QIIME2 software, the number of observed OTUs and the Chao 1 index were measured to estimate α**-**diversity, and principal coordinate analysis (PCoA) was used for estimating β-diversity (28). All graphs and diagrams were constructed with the ggplot2 package in R (ver. 4.1.2). The Wilcoxon rank-sum test was used for comparisons between groups. The PICRUSt2 tool was used for predicting the metabolic functions of bacteria (29), where the metabolic functions were estimated by mapping the composition of the identified bacteria into the KEGG database. To identify different metabolic functional abundances between groups, Statistical Analysis for Metagenomic Profiles (STAMP) was used (30) with a Bonferroni correction-adjusted P value < 0.01 as a significance cutoff.

### Statistical analysis

Continuous variables were tested for normality and then analyzed using Student’s t test or the Mann-Whitney U test. The data are expressed as means ± standard deviations (SD) or median with interquartile range (IR). The relative abundance of each taxon was calculated through total sum normalization methods. Nonparametric statistical analyses such as the Mann-Whitney test and Kruskal-Wallis test were performed to compare the relative abundance of taxa and α-diversity of each group using Prism 8 (GraphPad Software, Inc., San Diego, CA, USA). A permutational multivariate analysis of variance (PERMANOVA) with 999 permutations for pairwise tests was carried out to analyze the β-diversity of each group using qiime2-2021.8 and silva 13899 similarity. In all analyses, P < 0.05 indicates statistical significance.

## Results

### Characteristics of enrolled axSpA patients

A total of 33 axSpA patients and 20 HCs were included. Thirty-two of 33 axSpA patients were positive for human leukocyte antigen (HLA)-B27 and 51.5% used a TNF inhibitor. Mean age and percentage of males were higher in patients with axSpA than in HCs (P = 0.02 and P = 0.01, respectively). Other information is summarized in **Table 1**.

**Table 1:** Patient demographics and disease related parameters.

	Healthy controls (N = 20)	axSpA (N = 33)	*P*
Age (years)	33.0 ± 5.7	42.3 ± 12.4	0.02
Gender			0.01
Female	16 (80%)	3 (9.1%)	
Male	4 (20%)	30 (90.9%)	
Disease duration (months)	–	106.0 ± 88.6	
BMI (kg/m^2^)	–	23.8 ± 3.4	
AS or nr-axSpA	–		
AS		21 (63.6%)	
nr-axSpA		12 (36.4%)	
TNFi use	–	17 (51.5%)	
NSAID use	–	26 (78.8%)	
PGA	–	3.0 ± 2.2	
BASDAI	–	2.9 ± 2.1	
ASDAS-CRP	–	1.8 ± 0.9	
ASDAS-ESR	–	1.7 ± 0.9	
ESR (mm/hr)	–	8.1 ± 11.3	
CRP (mg/dL)	–	0.3 ± 0.6	
HLA-B27 positive	–	32 (97.0%)	

ASDAS, Ankylosing Spondylitis Disease Activity Score; BASDAI, Bath Ankylosing Spondylitis Disease Activity Index; BMI, body mass index; HTN, hypertension; DM, diabetes mellitus; ESR, erythrocyte sedimentation rate; CRP, C-reactive protein; TNF, tumor necrosis factor; NSAID, non-steroidal anti-inflammatory drug; PGA, patient’s global assessment.

### Comparison of gut microbiome compositions between patients with axSpA and HCs

**Figure 1:**
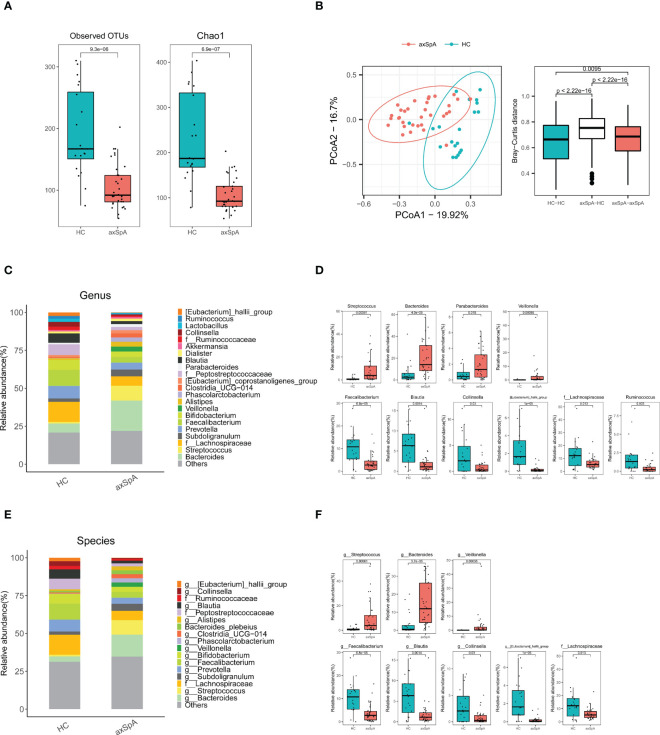
Comparison of gut microbiomes between axial spondyloarthritis (axSpA) patients and healthy controls (HCs). Feces were obtained from axSpA patients (N = 33) and HCs (N = 20), and 16s rRNA was analyzed using next generation sequencing. **(A)** α-diversity measured by observed OTUs and Chao1 score and **(B)** β-diversity using PCoA based on Bray-Curtis dissimilarity between axSpA patients (N = 33) and HCs (N = 20). Pearson’s correlation (r) and the corresponding *P* values are presented. Relative abundance of gut microbes in axSpA patients and HCs according to genus **(C, D)**, and species levels **(E, F**). Species-level gut microbes that were significantly different between groups are presented. OTUs, operational taxonomic units; PCoA, principal coordinate analysis.

Patients with axSpA had significantly different microbial diversity and composition compared to the HC group. As shown **Figure 1**, the number of observed OTUs and Chao1 index measuring α-diversity was smaller in the axSpA group (**Figure 1A**), while PCoA plots for estimating β-diversity indicated that the bacterial communities in axSpA samples were significantly different from those of HCs (P < 0.01) (**Figure 1B**, left panel). In addition, the estimation of Bray-Curtis dissimilarity showed that microbial communities showed significantly more diversity between groups than within groups (**Figure 1B**, right panel).

We then examined differences in microbial compositions according to taxonomical rank from phylum to species. For instance, at the phylum level, the Bacteroidota and Proteobacteria phyla were abundant in axSpA patients, whereas the Firmicutes phylum was abundant in HCs (**Supplementary Figures 1A, B**). At the family level, Streptococcaceae, Bacteroidaceae, Enterobacteriaceae, and Veillonellaceae were abundant in axSpA patients, whereas Ruminococcaceae, Lachnospiraceae, Coriobacteriaceae, and Erysipelotrichaceae were abundant in HCs (**Supplementary Figures 1C, D**). At the genus level, Bacteroides and Faecalibacterium emerged to be the second most enriched bacteria in axSpA and HCs, respectively (**Figures 1C, D**). At the species level, Bacteroides (g) and Streptococcus (g) were significantly more abundant in axSpA patients than in HCs (**Figures 1E, F**), whereas F. prausnitzii, Blautia (g) was significantly more abundant in HCs than in axSpA patients (**Figures 1E, F**). Among the microbial species that differed significantly between the two groups, only Veillonella (g) showed a positive correlation with disease activity score (ASDAS-CRP) (r = 0.37, P = 0.03) (**Supplementary Figure 2**).

### Comparison of microbial functional pathway analysis between axSpA and HC groups

**Figure 2:**
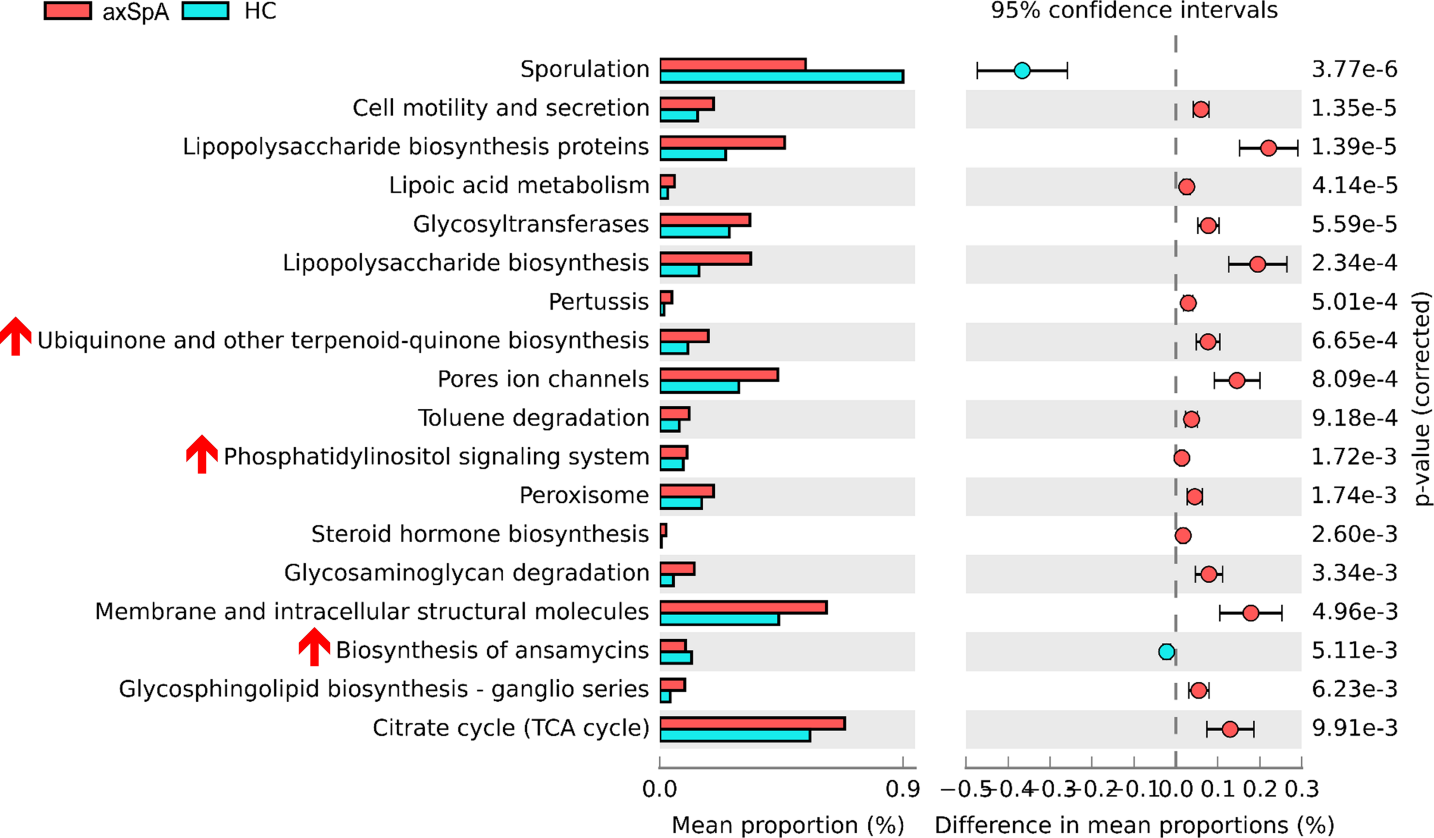
Microbial pathway analysis between axSpA patients and HCs. Functional pathway predictions with bacteria from axSpA patients and HCs. KEGG pathway analysis of OTUs enriched differentially between axSpA patients and HC microbiomes were analyzed using PICRUSt. *P* values are based on Welch’s test.

We then predicted microbiome-driven metabolic functions using the PICRUSt2 tool. We observed that enrichments of several microbial pathways differed significantly between the axSpA and HC groups (**Figure 2**), suggesting that abnormal changes in microbial composition may be associated with the pathogenesis of axSpA. For instance, several inflammation-associated pathways, including cell motility, secretion, and lipopolysaccharide biosynthesis proteins; glycosyltransferases (31); glycosphingolipid biosynthesis (32); and the citrate cycle (33), were more abundant in axSpA patients than in HCs. Surprisingly, some anti-inflammatory pathways, such as ubiquinone biosynthesis (34) and phosphatidylinositol signaling (35), were also more abundant in the axSpA group. The subgroup analysis between axSpA with or without TNFi showed that pentose/glucuronate interconversions, galactose metabolism, pentose phosphate pathway, and phenylpropanoid biosynthesis were relatively more abundant in axSpA patients who did not use TNFi than axSpA patients who used TNFi (**Supplementary Figure 3**).

### Impact of F. prausnitzii or butyrate on CD4^+^ T cell differentiation and cytokine production

**Figure 3:**
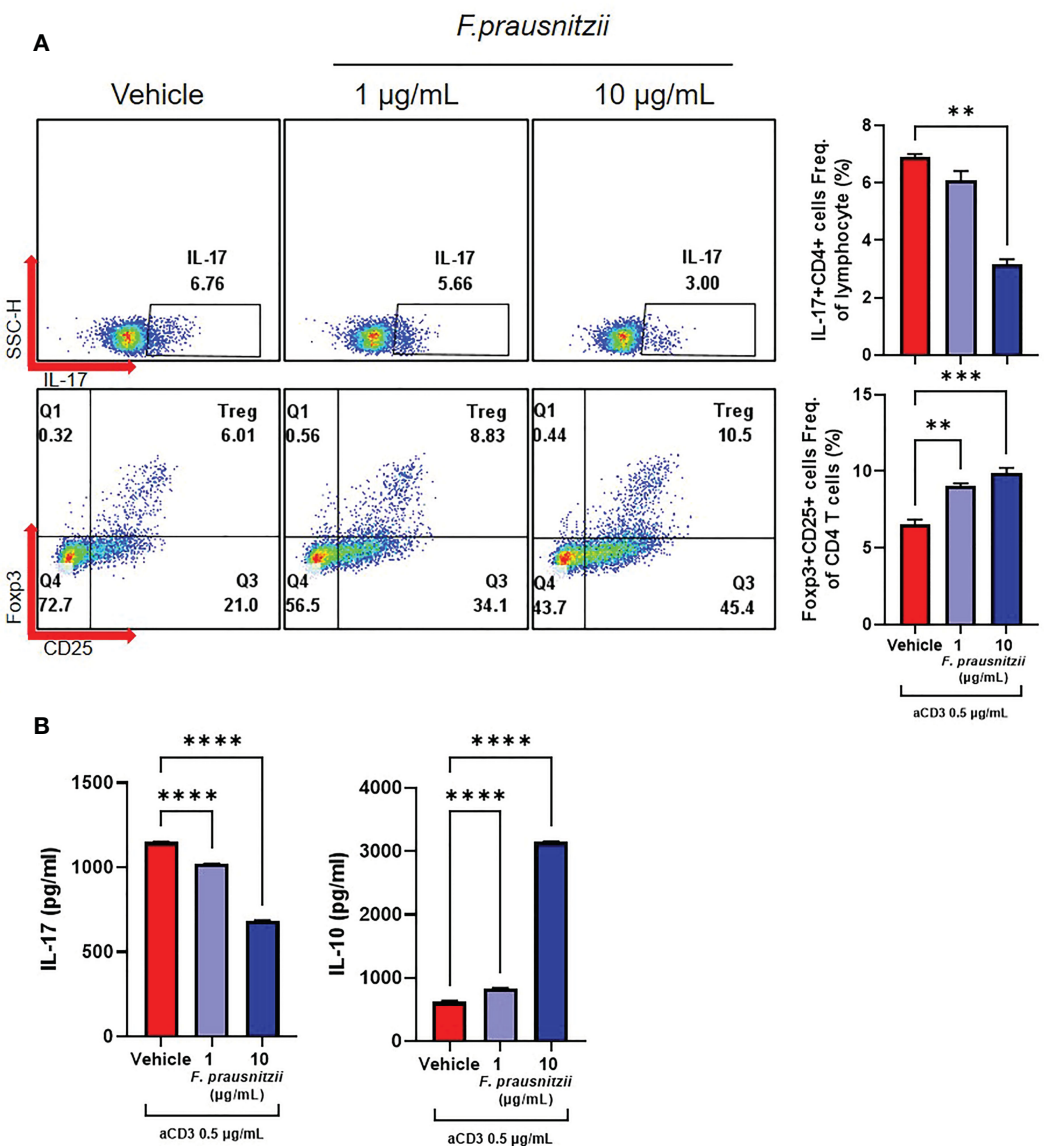
Effects of *Faecalibacterium prausnitzii* on the CD4^+^ T cell differentiation of axSpA patient-derived PBMCs. PBMCs (1 x 10^6^ cells) were obtained from axSpA patients (N = 4). These were cultured with *Faecalibacterium prausnitzii* (1 or 10 μg/mL) with anti-CD3 Ab (0.5 μg/mL) for 72 h. **(A)** CD4^+^ IL-17A^+^ T cell and CD4^+^ CD25^high^ Foxp3^+^ T cell differentiation was measured by flow cytometry (presented with representative flow cytometry image). **(B)** In culture media, the levels of IL-17A and IL-10 were measured by ELISA. ^**^ *P* < 0.01, ^***^ *P* < 0.001 and ^****^ *P* < 0.0001.

**Figure 4:**
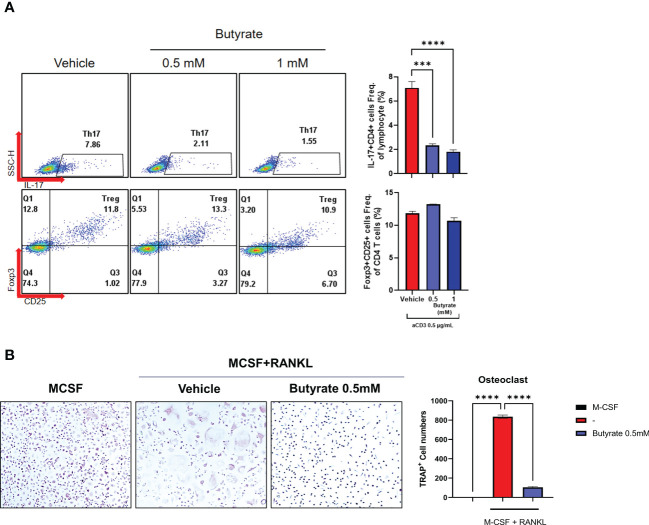
Effects of butyrate on the CD4^+^ T cell differentiation and osteoclastogenesis of axSpA patient-derived PBMCs. **(A)** PBMCs (1 x 10^^6^ cells) were obtained from axSpA patients (N = 4). These were cultured with butyrate (0.5 or 1 mM), with anti-CD3 Ab (0.5 μg/mL) for 72 h. CD4^+^ IL-17A^+^ T cell and CD4^+^ CD25^high^ Foxp3^+^ T cell differentiation was measured by flow cytometry (presented with representative flow cytometry image). **(B)** PBMCs (1 x 10^6^ cells) from axSpA patients were cultured with M-CSF (30 ng/mL) and RANKL (100 ng/mL) with vehicle or butyrate 0.5 mM for 10–14 days. The TRAP^+^ multinucleated cells were counted. ^***^ *P* < 0.001, ^****^ *P* < 0.0001.

PBMCs and CD4^+^ T cells derived from axSpA patients (N = 4) were cultured under the administration of F. prausnitzii or butyrate with anti-CD3 (0.5 μg/mL). When F. prausnitzii was added to PBMCs of axSpA, decrease of CD4^+^ IL-17A^+^ T cell population (F. prausnitzii 10 μg/mL) and increase of CD4^+^ CD25^high^ Foxp3^+^ T cell population (F. prausnitzii 1, 10 μg/mL) were observed (**Figure 3A**). Also, the level of IL-17 decreased and levels of IL-10 increased in culture media of PBMC culture (**Figure 3B**). In CD4^+^ T cell purified experiment, the CD4^+^ IL-17A^+^ T cell population was lower in the F. prausnitzii 5 μg/mL treatment group compared to the control, whereas CD4^+^ CD25^high^ Foxp3^+^ T cells population did not change by F. prausnitzii (**Supplementary Figure 4A**). The levels of IL-17A in culture media were downregulated in the F. prausnitzii 10 μg/mL treatment groups, and IL-10 was increased in the F. prausnitzii 1 μg/mL and 10 μg/mL groups (**Supplementary Figure 4B**). CD4^+^ IL-17A^+^ T cell population decreased by butyrate in PBMC culture (**Figure 4A**). The addition of butyrate 0.5 mM downregulated the CD4^+^ IL-17A^+^ T cell population and CD4^+^ CD25^+^ Foxp3^+^ T cell population in CD4^+^ T cell purified experiment (**Supplementary Figure 5**). Similarly, TRAP^+^ multinucleated cell counts were significantly lower in the butyrate 0.5 mM + M-CSF + RANKL treatment group than in the M-CSF + RANKL group (**Figure 4B**).

### Anti-arthritic effects of butyrate in a curdlan-induced spondyloarthritis mouse model

**Figure 5:**
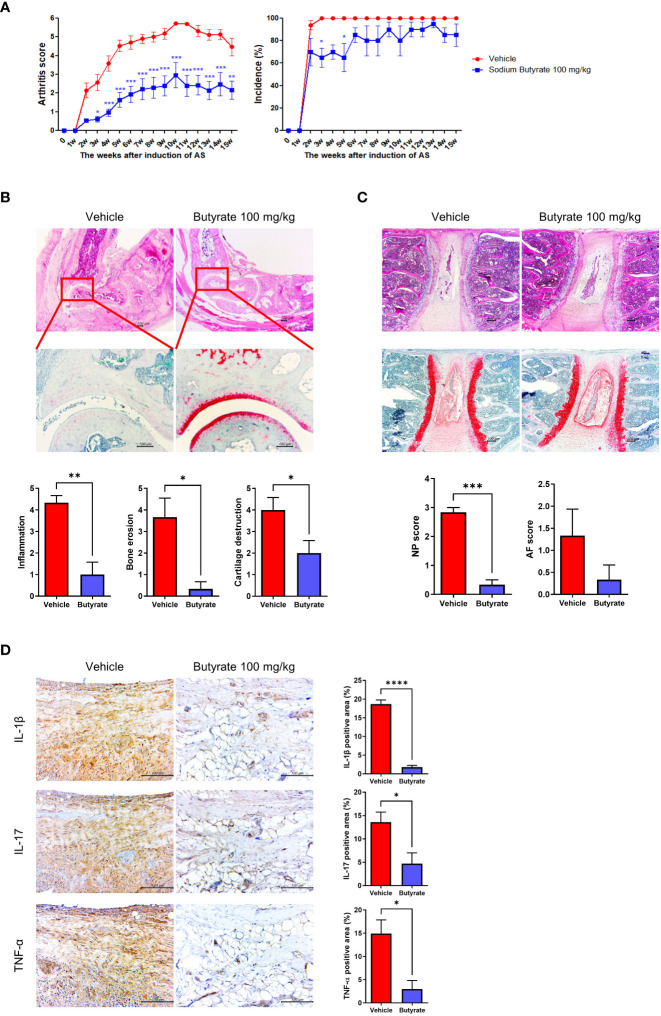
Effects of butyrate treatment on curdlan-induced SpA mice. SKG mice were injected with 3 mg of curdlan through intraperitoneal and oral administration. One week later, sodium butyrate (100 mg/kg) was administered orally once a day for 14 weeks. **(A)** Arthritis score and incidence were monitored by observation twice a week. **(B, C)** At 15 weeks post curdlan injection, joint and spine tissue samples were collected from all groups. Histopathology was evaluated for H&E and Safranin O staining. Inflammation, bone erosion, cartilage destruction, and nucleus pulposus (NP) and annulus fibrosus (AF) scores were assessed in both groups. **(D)** Synovial tissues in joints were immunohistochemically stained for IL-1β, IL-17, and TNF-α. Positive area (%) was analyzed with the color deconvolution tool in ImageJ. ^*^ *P* < 0.05, ^**^ *P* < 0.01, ^***^ *P* < 0.001 and ^****^ *P* < 0.0001.

Curdlan-immunized SKG mice were treated with or without butyrate 100 mg/kg/day for 15 weeks (N = 5 for each group). The arthritis score was significantly lower in the butyrate-treated group, but the incidence rate of arthritis was similar between the two groups (**Figure 5A**). On H&E and Safranin- O staining of the hind feet, the degree of inflammation, erosion, and cartilage destruction were diminished in butyrate-treated mice (**Figure 5B**). in addition, NP and AF scores were downregulated in butyrate-treated group (**Figure 5C**). Proinflammatory cytokines (IL-1β, IL-17, TNF-α)-expressing cells were decreased in the butyrate-treated group (**Figure 5D**).

**Figure 6:**
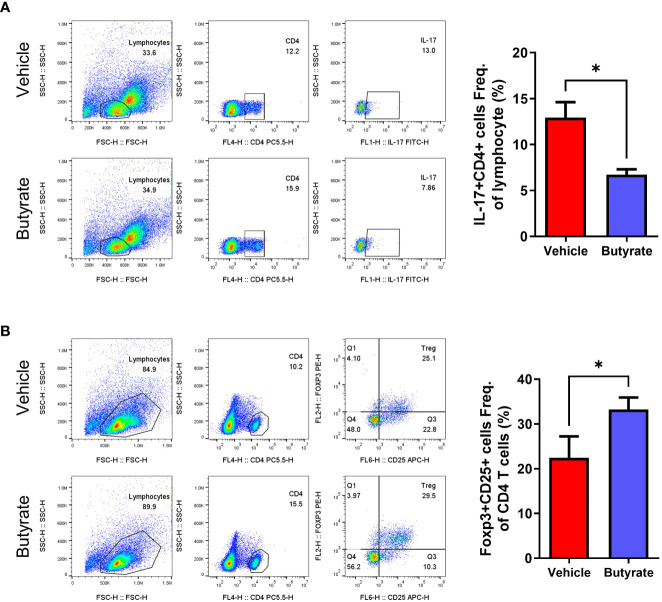
Effects of butyrate on the CD4^+^ T cell differentiation of SpA mice splenocytes (*ex vivo*). At 15 weeks post curdlan injection, **(A, B)** The expression of Th17 (CD4^+^IL-17^+^), and Tregs (CD4^+^CD25^+^ FOXP3^+^) in total CD4^+^ cells of splenocytes were assessed using flow cytometry (presented with representative flow cytometry image). ^*^ *P* < 0.05.

Splenocytes were harvested from the butyrate-treated and non-treated groups at 15 weeks after curdlan injection, then flow cytometry were performed to examine the IL-17A^+^ CD4^+^ and CD25^+^ Foxp3^+^ CD4^+^ T cell populations. The results showed significant decrement of the IL-17A^+^ CD4^+^ T cell population in the butyrate-treated group (**Figure 6A**). The CD25^+^ Foxp3^+^ CD4^+^ T cell population was increased in the butyrate-treated group (**Figure 6B**).

Splenocytes were next obtained from SKG mice not immunized with curdlan and cultured under Th17 polarizing conditions. The *in vitro* results demonstrated a decrease in the IL-17A^+^ CD4^+^ T cell population and in the levels of IL-17A and TNF-α in culture media in the butyrate-treated group (**Supplementary Figure 6**).

## Discussion

In the present study, we demonstrated the difference in gut microbiome diversity and composition between axSpA patients and HCs. This was the first study of its kind conducted in Korean axSpA patients. Similar to previous studies, the α-diversity of axSpA patients was decreased compared to HCs, and the composition of gut microbiota differed between the two groups. In particular, butyrate-producing bacteria were decreased in the feces of axSpA patients. In addition, we demonstrated the potential beneficial effects of butyrate and SCFA-producing bacteria such as *F. prausnitzii* through their ability to regulate pathological helper T cells. The osteoclastogenesis of axSpA patient-derived cells was decreased by butyrate administration, as was the arthritis score, proinflammatory cytokine expression in affected joints, and Th17 cell population in the SpA mouse model.

Gut dysbiosis in axSpA is well known. Previous studies have consistently shown decreased gut microbiome diversity in axSpA patients compared to HCs, however, the specific microbiome composition results vary (9). A decrease in *F. prausnitzii* has been shown previously, in two studies examining enthesitis-related arthritis in SpA patients (8, 36), whereas a quantitative metagenomic analysis of AS patients demonstrated an opposite result (37). *F. prausnitzii* is a well-known gut microbe that produces butyrate, which was suggested to be an anti-inflammatory mediator with therapeutic effects in a colitis animal model and intestinal epithelial cells (13, 38). The family *Lachnospiraceae* is another butyrate-producing bacteria (39), and the genus *Blautia* is known to produce other SCFAs (40). Decreases in *F. prausnitzii, Lachnospiraceae*, and *Eubacterium* were similarly observed in other microbiome data from axSpA patients (8, 36, 37, 41).

In the present study, we demonstrated that *F. prausnitzii, Lachinospiracea*, and *Balutia* were decreased in axSpA patients as well as the *in vitro* regulatory role of *F. prausnitzii* on Th17, Tregs, proinflammatory cytokine IL-17A, and immunoregulatory cytokine IL-10. *F. prausnitzii* treatment has been tried in several animal and preclinical studies of obesity-related or radiation therapy-related gut disorders, where it showed beneficial anti-inflammatory effects (42–44). Still, debate exists on whether gut dysbiosis causes or results from axSpA pathogenesis (45). In psoriatic arthritis, the administration of probiotics attenuated disease activity and recovered the tight junction of the intestine (46). In addition, butyrate treatment reduced arthritis severity, which was accompanied by a reduction in pro-inflammatory cytokine expression and Th17 in curdlan-induced SpA mice. A previous study using HLA-B27/β2m transgenic rats showed that propionate, but not butyrate, had anti-arthritic effects (14). Our study shows the potential pathological role of gut dysbiosis in Korean axSpA patients, and the potential of *F. prausnitzii* and its metabolite butyrate as a therapy in this population.

Several previous studies have tried to reveal the role of the gut microbiome as a biomarker for axSpA, and some specific microbiome showed correlation with disease activity of axSpA patients (47–49). Our study showed that the relative abundance of *Veillonella* correlated with ASDAS-CRP scores in axSpA patients. Participants in previous studies showed a lower percentage of biologics users (30.6–44.7%) than in our study (51.5%), whereas the disease activity score of axSpA patients enrolled in previous studies showed higher score than present study (47–49). SpA medication can affect the composition of the gut microbiome, and TNFi treatment has been shown to restore it to a eubiotic state in SpA patients (41, 50, 51). In the present study, the relative abundance of *F. prausnitzii* in TNFi users was higher than in TNFi non-users, but the difference was not significant (data not shown). The mean ASDAS-CRP score in axSpA patients enrolled in the present study was 1.8, which implies that most of them were in a stable, inactive stage of the disease. Unfortunately, data on the differences in the gut microbiome before and after TNFi treatment were not accessible in the present study. In addition, the composition of the gut microbiome is influenced by various factors such as age, sex, ethnicity, diet, and method of sample acquisition (52, 53). Therefore, to identify reliable microbiome biomarkers, future studies with a larger sample size including various ages, sex, and ethnicities with controlled diet and medications are required.

To access not only the differences in gut microbiome composition between axSpA patients and HCs but also the functional pathways dominantly expressed in axSpA, we used the PICRUSt2 tool. Several inflammation-associated microbial pathways, such as cell motility and lipopolysaccharide synthesis, as well as some anti-inflammatory pathways, were upregulated in the axSpA group. These results cannot conclude which microbial pathway has causative pathologic effects on axSpA pathogenesis. Furthermore, the present results cannot conclude whether these altered microbial pathways are a causative factor, result, or by-stander of axSpA pathogenesis.

Th17 and Treg populations have been suggested as biomarkers for treatment responses in axSpA. The circulating Th17 population is increased (4), whereas the Treg population is decreased (54), in patients with AS, which is a prototype of axSpA. In axSpA patients, TNFi responders showed increased Treg and decreased Th17 populations after treatment, whereas non-responders did not (5). IL-17A is the main pathologic Th17 cytokine (3), and IL-10 is the main immunoregulatory cytokine of Tregs (55). The present study revealed the regulatory role of *F. prausnitzii* and butyrate on Th17/Treg differentiation and IL-17A/IL-10 production *via in vitro* experiments. Butyrate treatment also reduced other proinflammatory cytokines such as IL-1β and TNF-α in SpA mice. These results could be the basis of future *in vivo* studies on the therapeutic role of *F. prausnitzii* and butyrate in axSpA treatment. Furthermore, the regulation of CD4^+^ T cell differentiation was more prominent in butyrate treatment than *F. prausnitzii* which suggest product of microbiome (butyrate) may have higher possibility as future therapy in axSpA than specific microbiome.

Abnormally increased osteoblastic activity and formation of irreversible bony ankylosis of spinal and sacroiliac joints are the hallmark of pathologic joint lesions in axSpA (1, 56, 57); that said, reduced bone mineral density of spongy bone and the consequently increased risk of osteoporosis is also observed in axSpA patients (58). Proinflammatory cytokines that are increased in axSpA patients, such as TNF-α, IL-17, and IL-22, result in excessive osteoclast differentiation compared to HCs (59, 60), and proper control of osteoclastogenesis is another important treatment goal in axSpA. In the present study, the administration of butyrate in axSpA patient-derived cells reduced osteoclast formation, and this implies that SCFAs may have the potential to regulate the inflammatory response (reduction of proinflammatory cytokines, helper T cell modulation) and reduce excessive osteoclastogenesis in axSpA.

There are several limitations in the present study. First, the sample size was small. Second, important factors affecting the gut microbiome, including medication (i.e., TNFi) and diet, were not strictly controlled. Third, we demonstrated the *in vitro* effects of *F. prausnitzii* and butyrate on CD4*^+^* T cell differentiation and the *in vivo* effects of butyrate in SpA mice; however, *in vivo* effects in axSpA patients should be evaluated in the future. Also, there was some discrepancies in aspect of regulating CD4^+^Foxp3^+^CD25^+^ T cell population between human CD4^+^ T cell, human PBMC, and mice splenocytes by butyrate or *F. prausnitzii*. The population of CD4^+^Foxp3^+^CD25^+^ T cell increased in axSpA patients’ driven PBMC after *in vitro* administration of *F. prausnitzii* (**Figure 3A**) and *ex vivo* analysis of splenocytes from curdlan induced SpA mice of butyrate treatment group (**Figure 6B**), no significant change in purified CD4^+^ T cell from axSpA patients after *in vitro* administration of *F. prausnitzii* (**Supplementary Figure 4A**) and *in vitro* administration of butyrate in axSpA patients’ PBMC (**Figure 4A**), and decreased *in vitro* administration of butyrate on CD4^+^ T cell of axSpA patients (**Supplementary Figure 5**). Future studies measuring changes in gut microbiota and disease activity before and after treating with specific potential probiotics or their metabolites in axSpA patients could prove the potential of modulating gut dysbiosis as a therapeutic option in axSpA. Fourth, disease activity in the enrolled axSpA patients was relatively low, so our results may not reflect the active disease state of axSpA. Fifth, we only evaluated the impact of *F. prausnitzii* and butyrate on CD4^+^ T cell differentiation and cytokine production, and other microbiota or SCFAs may have different effects. Sixth, the sex ratio and age of axSpA group and HC group differed: male dominance in axSpA group, whereas female dominance in HC group, and higher mean age in axSpA group than HC group. Finally, sample size of blood obtained from axSpA patients (N = 4) was relatively small than number of stool sample (N = 33). Invasive procedure (needling) is required to get blood sample, and the blood samples were only obtained from axSpA patients who agreed with the blood sampling.

In conclusion, the gut microbiome diversity and composition of axSpA patients differed from HCs, and this was the first study focusing on Korean axSpA patients. Several butyrate-producing taxa were decreased in axSpA patients, and their microbial pathways were altered, compared to HCs. The administration of *F. prausnitzii* or butyrate regulated Th17/Treg imbalances, and cytokine production, arthritis scores, proinflammatory cytokine expression, and Th17 differentiation were attenuated by butyrate treatment in SpA mice. These results imply the beneficial potential of restoring *F. prausnitzii* or butyrate in axSpA patients.

## Data availability statement

The datasets presented in this study can be found in online repositories. The names of the repository/repositories and accession number(s) can be found in the article/**Supplementary Material**.

## Ethics statement

The experimental protocol was approved by the Institutional Review Boards of Seoul Saint Mary’s Hospital (KC18TESI0627) and Konkuk University Medical Center (KUH 2019–08–015). Written informed consent was obtained from each participant prior to enrollment. The patients/participants provided their written informed consent to participate in this study. All animal research was conducted in accordance with the Laboratory Animals Welfare Act, the Guide for the Care and Use of Laboratory Animals, and the Guidelines and Policies for Rodent Experimentation provided by the Institutional Animal Care and Use Committee (IACUC) at the School of Medicine of The Catholic University of Korea (Approval number: CUMS–2020–0312–03). Written informed consent was obtained from the individual(s) for the publication of any potentially identifiable images or data included in this article.

## Author contributions

HM, HN, JJ, and M-LC: Conception and design of study; HM, HN, JJ, JSL, IU, and SeoYL: Acquisition of data; HM, HN, JJ, SeoYL, SC, GP, HS, T-SS, Y-KK, JJL and S-KK: Analysis and interpretation of data; HM, HN and M-LC: Drafting the article; HM, HN and M-LC: Revising the article critically; M-LC and S-HP acquired funding. All authors contributed to the article and approved the submitted version.
