# Supplementary material for: Identification of gut dysbiosis in axial spondyloarthritis patients and improvement of experimental ankylosing spondyloarthritis by microbiome-derived butyrate with immune-modulating function
Source: Front Immunol. 2023 Apr 18;14:1096565. doi: 10.3389/fimmu.2023.1096565 (PMC10152063; doi:10.3389/fimmu.2023.1096565)
Supplement: Supplementary file 1 [file DataSheet_1.docx]

**SUPPLEMENTARY FIGURES**

**
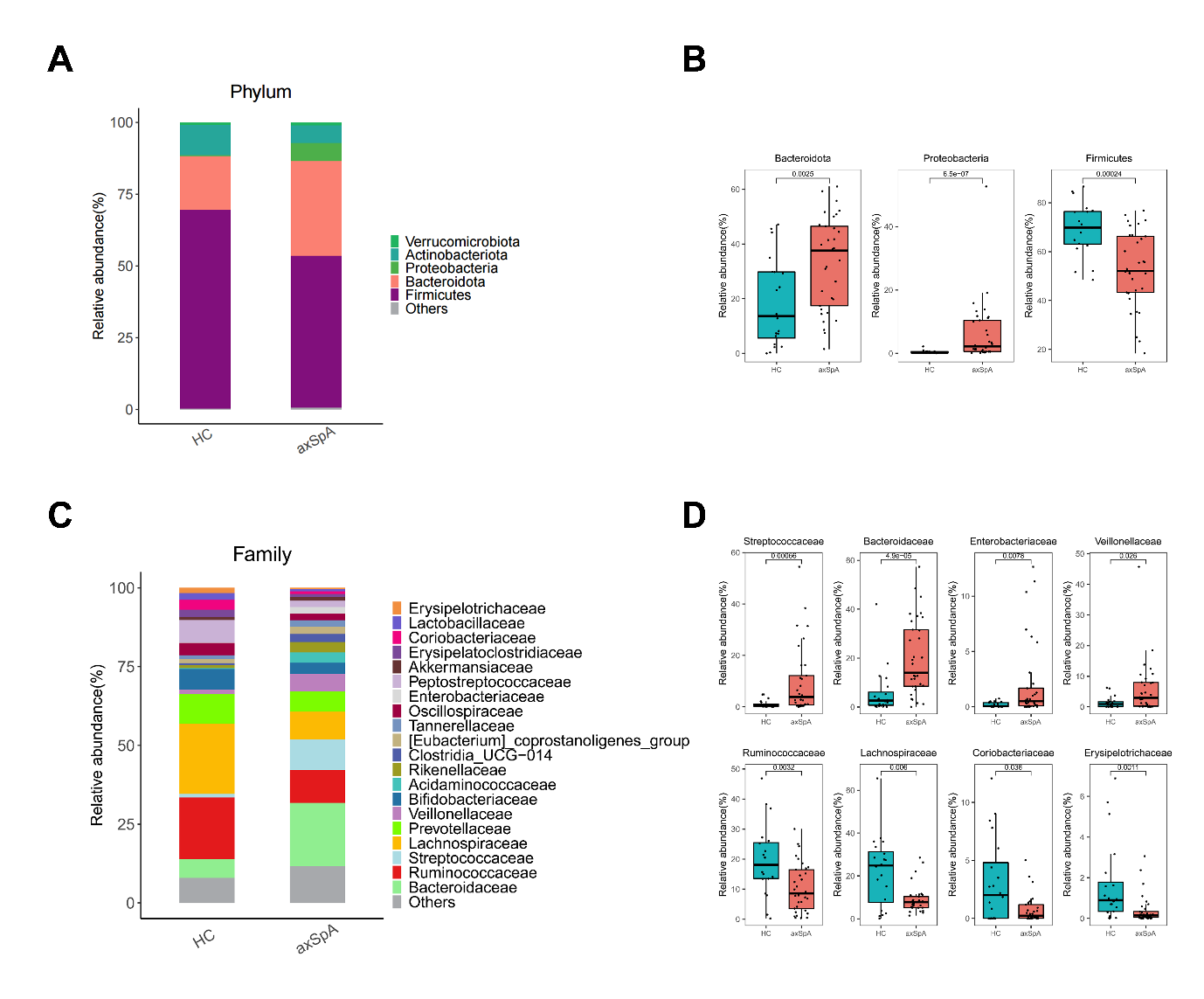
**

**Supplementary Fig. 1 Gut microbiome compositions in axSpA patients and HCs according to phylum, family levels. a, b.** Phylum level. **c, d.** family level. Significantly differed gut microbes according to phylum and family levels are presented. ^*^*P* < 0.05, ^**^*P* < 0.01, ^***^*P* < 0.001, and ^****^*P* < 0.0001.


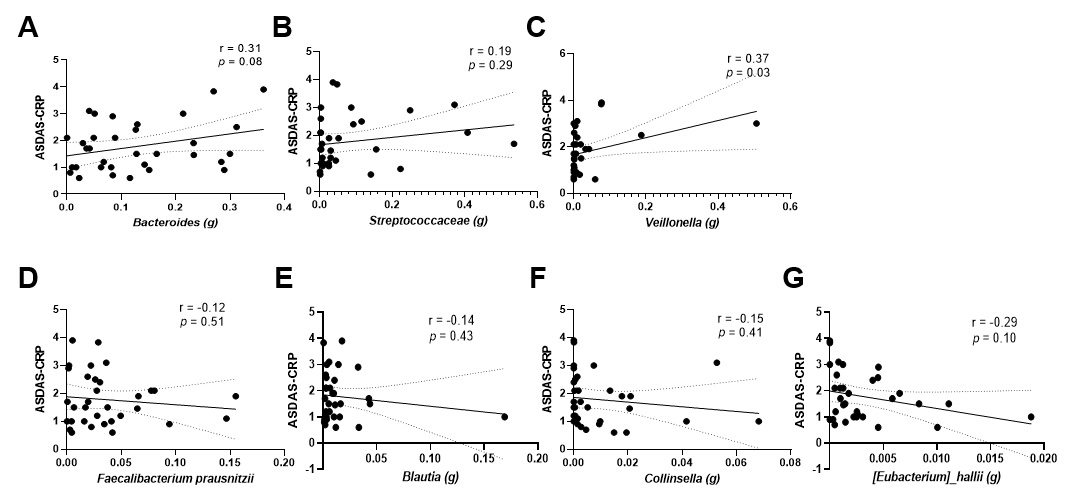


**Supplementary Fig. 2 Correlation between relative abundance and ASDAS-CRP score in axSpA patients.** Correlations between ASDAS-CRP score and *Bacteroides* (g) **a**, *Streptpcoccus* (g) **b**, *Veilonella* (g) **c**, *Faecalibacterium prausnitzii* **d**, *Blautia* (g) **e**, *Collinsella* (g) **f**, and *Eubacterium_halli* (g) **g**.

**
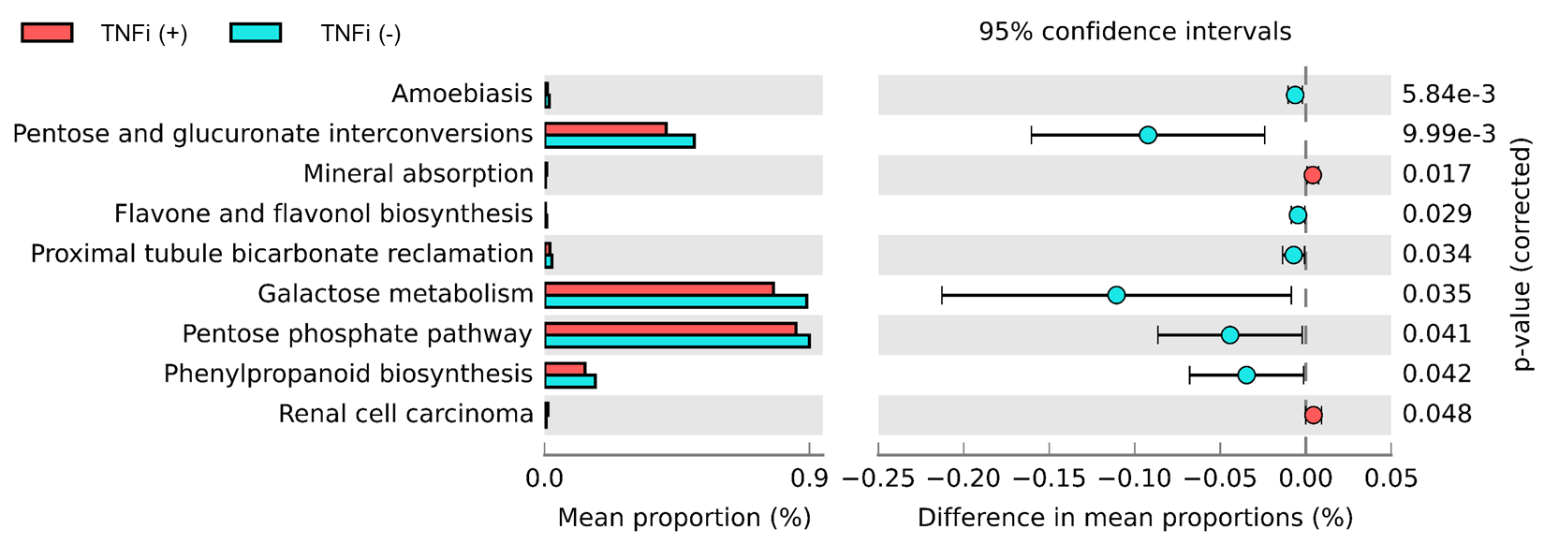
**

**Supplementary Fig. 3 Microbial pathway analysis between axSpA patients with TNFi and without TNFi.** Functional pathway predictions with bacteria from axSpA patients who used TNFi (TNFi [+], N = 17) and did not use TNFi (TNFi [-], N = 16). KEGG pathway analysis of OTUs enriched differentially between axSpA patients with or without TNFi microbiomes were analyzed using PICRUSt. *P* values are based on Welch’s test.

**
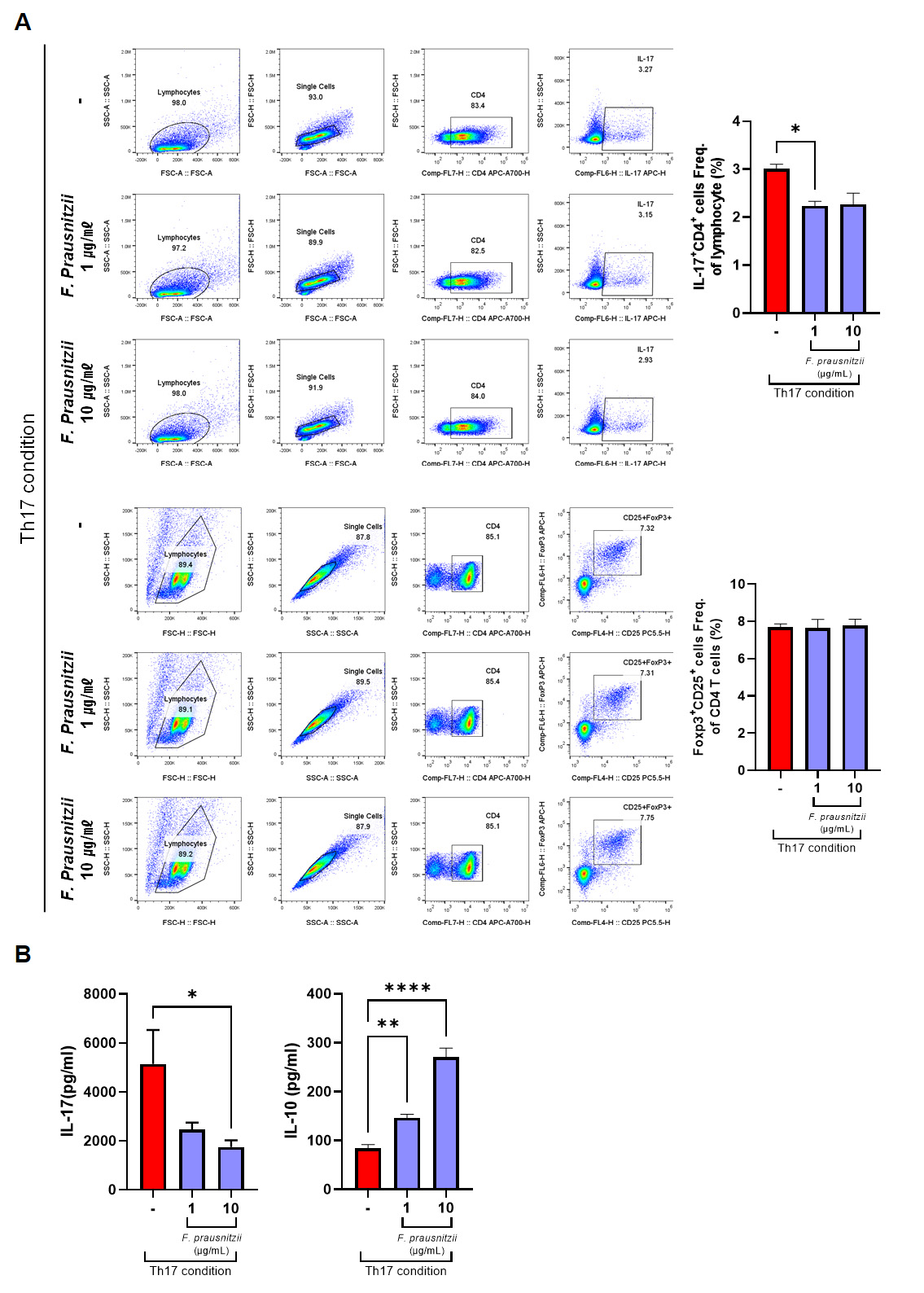
**

**Supplementary Fig. 4 Fig. 4 Effects of *Faecalibacterium prausnitzii* on the CD4^+^ T cell differentiation of axSpA patient-derived CD4^+^ T cells.** CD4^+^ T cells (5 x 10^5^ cells) were obtained from axSpA patients (N = 4). These were cultured with *Faecalibacterium prausnitzii* (1 or 10 μg/mL) with anti-CD3 Ab (0.5 μg/mL) for 72 h. **a.** CD4^+^ IL-17A^+^ T cell and CD4^+^ CD25^high^ Foxp3^+^ T cell differentiation was measured by flow cytometry (presented with representative flow cytometry image). **b.** In culture media, the levels of IL-17A and IL-10 were measured by ELISA. ^**^*P* < 0.01, ^***^*P* < 0.001 and ^****^*P* < 0.0001.

**
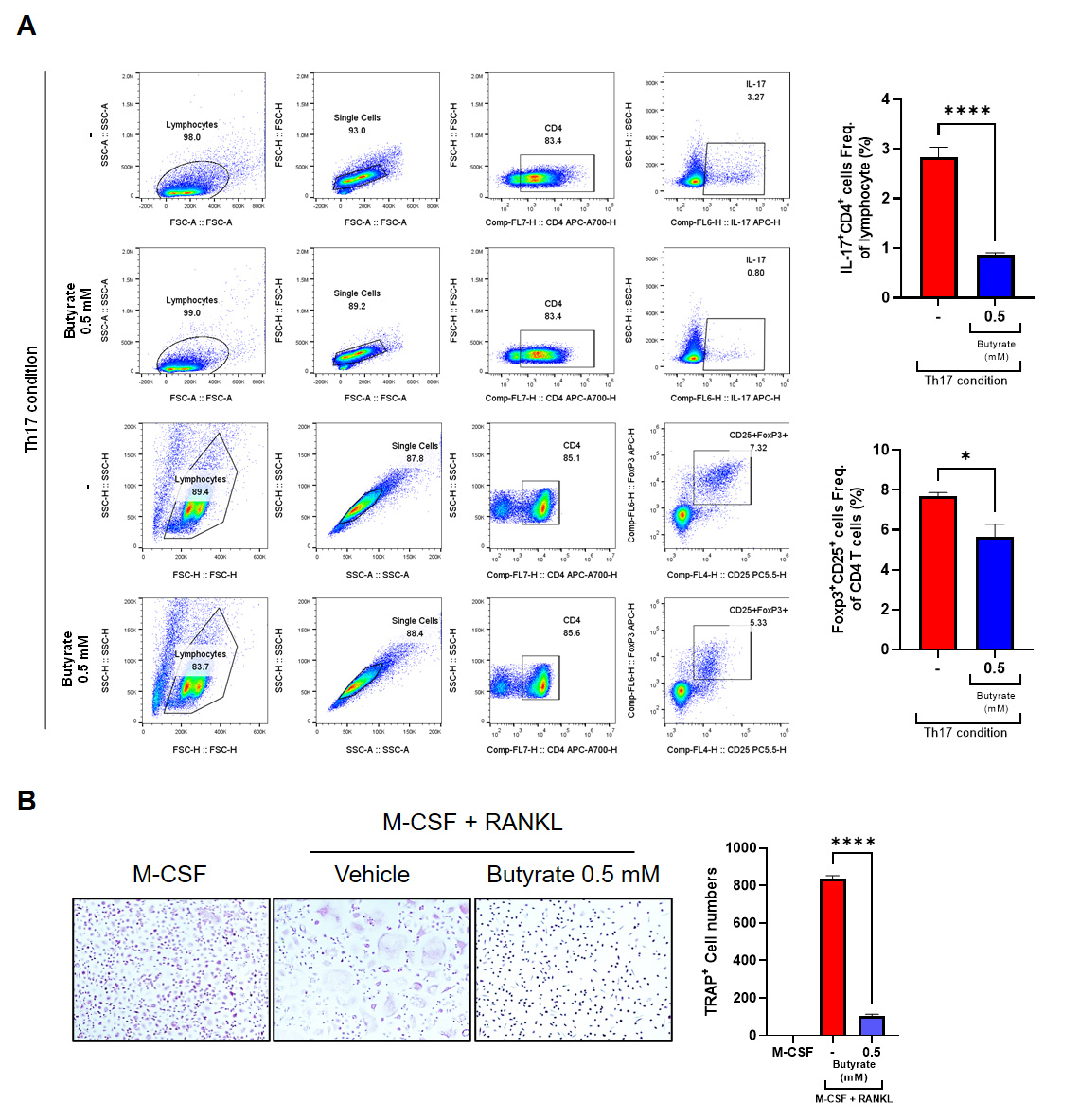
**

**Supplementary Fig. 5 Effects of butyrate on the CD4^+^ T cell differentiation of axSpA patient-derived CD4^+^ T cells.** CD4^+^ T cells (5 x 10^5^ cells) were obtained from axSpA patients (N = 4). These were cultured with butyrate (0.5 mM), with anti-CD3 Ab (0.5 μg/mL) for 72 h. CD4^+^ IL-17A^+^ T cell and CD4^+^ CD25^high^ Foxp3^+^ T cell differentiation was measured by flow cytometry (presented with representative flow cytometry image).

**
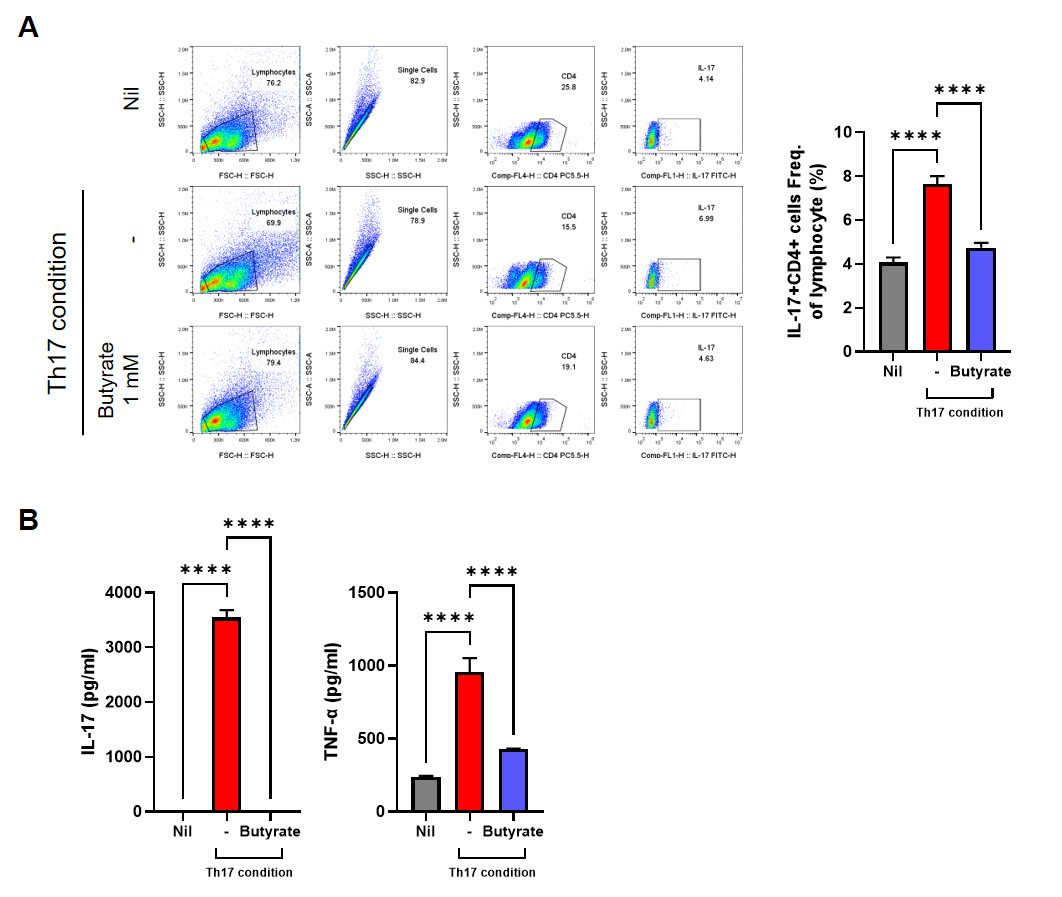
Supplementary Fig. 6 Effects of butyrate on CD4^+^ T cell differentiation in SKG mice splenocytes in vitro. a.** IL-17 expression in splenocytes from naïve SKG mice incubated for 3 days under Th17-polarizing conditions (stimulated only with anti-CD3 and anti-CD28 with TGF-β and IL-6). Flow cytometry images shown are representative flow cytometry images. **b.** Levels of IL-17 and TNF-α cytokines in supernatants were measured using ELISA. ^****^*P* < 0.0001.
